# Enzymatic Inhibitors from Natural Sources: A Huge Collection of New Potential Drugs

**DOI:** 10.3390/biom11020133

**Published:** 2021-01-20

**Authors:** Paolo Paoli

**Affiliations:** Dipartimento di Scienze Biomediche, Sperimentali e Cliniche Mario Serio, Università degli Studi di Firenze, 50134 Florence, Italy; paolo.paoli@unifi.it

For thousands of years, human beings have used natural products for the treatment of various types of pathologies. Even today, in many developing countries where modern medicines are still not available, many diseases are treated following the indications of traditional local medicine, using plant and algae extracts. Traditional remedies have the advantage of being well tolerated by people but have often shown little effectiveness, mainly because the natural extracts contain a limited dose of the required active molecule, or contain compounds acting as antagonists to it. Therefore, despite the evidence that traditional medicine represents an important resource for the treatment of human diseases, the availability of new potent, targeted and safe drugs represents one of the most important objectives in combating both acute and chronic diseases.

In recent decades, technological progress and the development of artificial intelligence have led many researchers to believe in the possibility of generating new drugs using only an in silico approach. However, this strategy proved to be unsuccessful in most cases. One of the main reasons for this failure is the limited human ability to imagine new scaffold molecules in comparison to the natural ones that world can offer. Plants, algae and microorganisms synthesize thousands of structurally different molecules, most of which are often not reproducible in modern organic chemistry laboratories. This evidence suggests that in the coming years, despite strong technological progress, drug discovery will still be highly dependent on the identification of new natural bioactive molecules that exhibit drug-like activity.

The purpose of this Special Issue is to confirm the enormous potential of the natural world in providing new molecules that exhibit different types of pharmacological activity. Thanks to the precious support of numerous researchers, this Special Issue has been enriched with fourteen original studies and two reviews.

The study of Phi Hung Nguyen et al. [1] aimed to evaluate the ability of some pimarane diterpenes from O. stamineus to inhibit PTP1B and stimulate glucose uptake in human adipocytes. The authors showed that some of such molecules behave as potent inhibitors of PTP1B, one the most important negative regulators of insulin receptor. Moreover, they showed that such molecules are not toxic, and stimulate glucose uptake in human adipocyte cells. Together, these pieces of evidence suggest that such molecules are interesting lead compounds to develop new antihyperglycemic agents.

The article by Ali S. Alqahtani et al. [2] explored the antidiabetic and antioxidant activities of plant extracts and their purified components from Nuxia oppositifolia, a plant on which aerial parts were used to obtain an extract with well-known antidiabetic properties. The authors demonstrated that the obtained extract contained two bioactive compounds, namely 3-oxolupenal and katononic acid, that showed antioxidant activities and behaved as inhibitors of α-amylase and α-glucosidase. Moreover, they determined the dissociation constant of compounds and investigated the interaction of 3-oxolupenal and katononic acid with these enzymes by in silico docking analysis, thereby shedding light on their mechanism of enzyme inhibition.

Najeeb Ur Rehman et al. [3] demonstrated that the methanolic extract of Boswellia elongata resin contains several triterpene acids that are able to inhibit α-glucosidase, showing IC50 values in the 9.9–56.8 µM range. The authors investigated the mechanism of action by kinetic and in silico docking analyses. Thanks to their efforts, the authors provided new interesting data about the mechanism of action of such natural compounds, highlighting that some of these behaved as allosteric inhibitors of α-glucosidase.

Auriane Dudoit et al. [4] and collaborators analysed biological properties of natural compounds extracted by Tannat-variety grapes. The authors evaluated the content in stilbenes, anthocyanins and flavan-3-ols in function of ripening stages, showing that these extracts showed an excellent in vitro inhibitory potential against α-glucosidase. Moreover, the authors studied the mechanism of action of extracts and investigated the role of various components in inhibiting α-glucosidase. Interestingly, they found that both anthocyanin and stilbene compounds are not involved while flavanols have a key role in the inhibition capacity of α-glucosidase.

With their study, Poonam Kalhotra et al. [5] explored the inhibitory activity of compounds derived from garlic on dipeptidyl peptidase-4, the serine protease that regulates glucose metabolism catalysing the degradation of glucagon-like peptides (GLP-1 and GLP-2) and glucose-dependent insulin releasing polypeptide GIP. By using mass spectrometry, the authors identified the caffeic acid 4-O-glucoside, malonylgenistin and calenduloside E as bioactive components of the garlic extract. Finally, using an in silico analysis they studied the interactions of these compounds with DPP-4, demonstrating that these were high affinity ligands. Altogether, these results provide new interesting insights into the biological activities of garlic extracts.

The results of a study conducted by F. Balestri et al. [6] showed that tea catechins act as differential inhibitors with respect to aldose reductase, an enzyme involved in the onset of diabetes-related pathological complications. Interestingly, the authors demonstrated that EGCG preferentially inhibited L-idose and GSHNE reduction with respect to HNE, while gallic acid was less active in inhibiting HNE and GSHNE reduction. Finally, they found that EGC was a less efficient inhibitor of aldose reductase and devoid of any differential inhibitory action. Together, these results define new interesting properties of tea components, and suggested that such molecules could contribute to prevent the onset of the diabetes-related pathologies.

The aim of the study conducted by Nabeelah Bibi Sadeer et al. [7] was to describe the biological properties of the phytochemical constituents of Bruguiera gymnorhiza (L.) Lam, a plant recommended for the treatment of various ailments, including diabetes and some associated pathologies. The authors used two solvents, methanol and ethyl acetate, and different parts of the plant, mainly leaves, roots, twigs and fruits, to obtain numerous extracts. These extracts were then analysed to determine their antioxidant and inhibitory power against a series of enzymes such as α-amylase, α-glucosidase, tyrosinase, elastase, lipase, AChE and BChE. The results of the multivariate analysis between the extraction solvents and the different parts of plants used showed that the choice of extraction solvents had a strong influence on the biological activities studied. In addition, the data reported in this study provided a comprehensive overview of the pharmacological potential of B. gymnorhiza.

The study conducted by Umar M. Badeggi et al. [8] aimed to evaluate antioxidant and antidiabetic properties of gold nanoparticles loaded with natural components from Leucosidea sericea. The nanoparticles, when conjugated with natural compounds, were able to inhibit both α-glucosidase and α-amylase, showing IC50 values in the low micromolar range and a relevant antioxidant activity. In conclusion, this study confirmed that it is relatively easy to generate bioactive gold nanoparticles for the delivery of natural compounds. Interestingly, the authors suggested that such nanoparticles could be used for treatment of different kind of pathologies, such as diabetes and inflammation.

The study conducted by Victor Marin et al. [9] demonstrated that natural drimane sesquiterpenoids such as isodrimeninol can be used as lead compounds to generate new potent antifungal drugs. The authors isolated the isodrimeninol from bark of Drimys winteri and used it as starting material for the synthesis of four new semisynthetic derivatives. Further analyses revealed that one of the compounds showed enhanced antifungal activity with respect to the original compound. Moreover, docking analyses revealed that this compound acts as a competitive inhibitor with respect to 14-α-demethylase. Taken together, these results showed that such a strategy can be successfully used to develop new drugs for the treatment of candidiasis.

The scope of the study performed by Ericsson Coy-Barrera [10] was to investigate the interaction of a custom-made library of 58 natural 2-arylbenzofuran-containing compounds with cyclooxygenase (COX) enzymes. Through molecular dynamics simulations and kinetic analyses, the author selected compounds exhibiting the most favourable binding energies and the lowest in vitro IC50 values for COX-2 inhibition. Moreover, 3D-QSAR analysis revealed the crucial structural requirements to optimize ligand binding and enzyme inhibition. In conclusion, this study allowed the characterization of four new natural compounds, suggesting that those could be used as main structures for the development of new COX-2 inhibitors.

The study of Thanh-Diep Ly et al. [11] aimed to identify natural compounds able to inhibit xylosyltransferase-I (XT-1), one of enzymes involved in the onset of fibroproliferative diseases. The initial screening, performed using a library of 96 natural compounds, allowed the identification of four compounds showing a significant inhibitory activity on XT-1. The binding of such molecules with the enzyme was also analysed by docking in silico, whereas their in vivo activities were confirmed by analysing their effects on human dermal fibroblasts. Interestingly, two of the selected compounds were also able to decrease the expression of XT-1 through molecular mechanisms involving both TGF-β and microRNA-21 signalling pathways.

Gabriel Zazeri et al. [12] presented the results of a deep investigation concerning the interaction of the natural compound piperine with interleukin-1 beta (IL-1β) using both experimental and computational molecular biophysical tools. The authors performed detailed analyses using fluorescence spectroscopy to evaluate the interaction mode and the affinity constant of piperine. Moreover, the authors investigated molecular interactions between the ligand and protein by in silico docking and molecular dynamic analyses. Data obtained give important information useful for supporting further drug discovery studies.

Behzad Shahin-Kaleybar et al. [13] focused their study on the identification and characterization of new cysteine-rich peptides from Citrullus colocynthis. Using specific extraction procedures and mass spectrometry, the authors identified 23 cysteine-rich peptides (CRPs) and 8 novel peptides. Detailed comparative analyses revealed the existence of minor variations with respect to known peptides, suggesting that those identified were novel subtypes of CRPs whose bioactivities have yet to be revealed.

The purpose of the study conducted by Xi Chen et al. [14] was to identify novel Bowman–Birk type trypsin inhibitors of the ranacyclin family. The authors, starting from the skin secretion of broad-folded frog (Sylvirana latouchii), identified an active peptide showing potent trypsin inhibitory activity and weak antimicrobial activity. The authors also generated two peptide analogues with improved antimicrobial and anticancer activities. Together, these pieces of evidence suggest that such peptides are excellent templates for the development of new multifunctional peptides to be used for prevention and cancer treatment.

Andrea Baier and Ryszard Szyszka [15] focused their attention on natural compounds the act as inhibitors of protein kinases. The enzymes belonging to this family control important cellular processes including proliferation, differentiation, apoptosis and cell metabolism. Their malfunction is often associated with devastating diseases, such as cancer, neurological disorders, and autoimmune diseases. For this reason, these enzymes are considered important pharmacological targets. In this study, the authors give an exhaustive and comprehensive view of natural compounds acting as protein kinase inhibitors, thereby confirming that the natural world offers an impressive range of active products that could be used as base molecules to develop new potent and selective drugs.

The study presented by Renata Tisi et al. [16] offers an exhaustive analysis on natural molecules still identified and characterized as inhibitors of the enzymes belonging to the family of Ras proteins. The activity of mutated/upregulated Ras proteins is essential to sustain growth of different types of cancer cells. For this reason, the Ras inhibitors represent one formidable weapon to fight tumour growth. As described by the authors, the natural world offers a huge number of molecules able to impair the synthesis, processing, activity and signalling of Ras proteins. Such evidence suggests that natural compounds represent the best source of inspiration for the development of new molecules able to inhibit the activity, or, in any case, influence the function of Ras proteins.

In conclusion, taken together the studies reported in this Special Issue confirmed that the natural world still represents an irreplaceable source of inspiration for all researchers that aim to identify new scaffold molecules for the synthesis of a new generation of drugs.
